# Seeking a Sufficient Data Volume for Railway Infrastructure Component Detection with Computer Vision Models

**DOI:** 10.3390/s23187776

**Published:** 2023-09-09

**Authors:** Alicja Gosiewska, Zuzanna Baran, Monika Baran, Tomasz Rutkowski

**Affiliations:** Nevomo IoT, 03-828 Warsaw, Poland; a.gosiewska@nevomo.tech (A.G.); z.baran@nevomo.tech (Z.B.); m.seniut@nevomo.tech (M.B.)

**Keywords:** object detection, computer vision, machine learning, railway

## Abstract

Railway infrastructure monitoring is crucial for transportation reliability and travelers’ safety. However, it requires plenty of human resources that generate high costs and is limited to the efficiency of the human eye. Integrating machine learning into the railway monitoring process can overcome these problems. Since advanced algorithms perform equally to humans in many tasks, they can provide a faster, cost-effective, and reproducible evaluation of the infrastructure. The main issue with this approach is that training machine learning models involves acquiring a large amount of labeled data, which is unavailable for rail infrastructure. We trained YOLOv5 and MobileNet architectures to meet this challenge in low-data-volume scenarios. We established that 120 observations are enough to train an accurate model for the object-detection task for railway infrastructure. Moreover, we proposed a novel method for extracting background images from railway images. To test our method, we compared the performance of YOLOv5 and MobileNet on small datasets with and without background extraction. The results of the experiments show that background extraction reduces the sufficient data volume to 90 observations.

## 1. Introduction

Nowadays, rail transportation is widely accessible and is one of the most popular travel forms worldwide. An increasing amount of studies show that trains are more environmentally friendly than cars [1,2,3]. Additionally, high-speed trains are serious competitors for air transport [4]; therefore, railway has a good prospect of development ahead of it. However, with the rapid growth comes a new set of challenges for rail management. The increased popularity will put more strain on the infrastructure, which will need to be inspected more often; what is more, the expanding rail connections network will result in more and more kilometers of track to monitor. All of this implies an increase in the labor workload related to infrastructure maintenance.

The need for track inspections stems from the fact that the track is susceptible to weather conditions, such as extreme temperatures, high levels of humidity, and air pollution. Studies show that broken rails and welds were the leading derailment cause on tracks in the United States [5]. However, any defect on the railroad track can carry immense costs and even lead to catastrophic incidents such as train derailments. That is why monitoring railway infrastructure is crucial for the safety of travelers and the reliability of public transportation.

Currently, infrastructure inspections are manual. The specially trained staff, based on the visual evaluation and measurements from dedicated devices, assess the degradation of the track. Such a procedure requires much human labor, translating into a high maintenance cost. Moreover, the inspection speed is limited to the efficiency of the human eye; thus, it requires time and is prone to mistakes [6]. The solution for the highlighted issues could be incorporating computer vision techniques, especially machine learning, in infrastructure monitoring. Machine learning is a branch of artificial intelligence that consists of algorithms that can optimize themselves based on the provided data.

Nowadays, machine learning models achieve human-level performance in many areas, including medicine, finance, and technology. Therefore, artificial intelligence algorithms can be successfully used to support human decisions, including visual monitoring of rail infrastructure [7]. In recent years, much research has been conducted on the usefulness of computer vision for railway applications. A variety of methods were successfully applied, starting from traditional pattern recognition [8], through classical machine learning models, such as support vector machines [9], k-nearest neighbors [10], or random forest [11], ending with deep neural networks [12,13,14,15]. The latter methods usually yield the best results due to their capacity to solve complex problems. Therefore, deep learning has great potential for detecting railway defects [16].

Computer vision-based infrastructure monitoring for fault identification usually consists of two steps: (1) detection of railway components and (2) component-specific identification of defects. It is worth noting that in step (2), different rail components require different machine learning models for fault detection. This is due to the specific characteristics of faults for different components, and therefore, many studies are focusing on only one component of the rail infrastructure. Studies include the detection of wheel defects [17,18], the identification of bolt corrosion [19,20], assessing ballast support for sleepers [21], aiding in the design of prestressed concrete railway sleepers [22], the recognition of rail surface cracks [23], capturing fastener defect detection [24], and monitoring bridges’ condition [25,26]. However, fault identification is impossible without accurate object detection (OD) in the previous step (1). For example, a crack on a sleeper would not be identified if the sleeper itself was not detected correctly. Due to its complexity and importance, separate studies often address the component-detection task, where deep neural networks detect track elements [27,28]. Over time, the need for rail object detection models will only increase. They will be in demand for various types of infrastructure elements and for different tracks, such as high-speed rail, maglev, or subway. Additionally, each country may need a different model due to the country-specific regulations and different ways the rail infrastructure is built. Therefore, it is important to study how to optimally build object-detection models.

This paper focuses on fast and accurate railway track component detection that can be used to support humans in monitoring rail infrastructure. We consider a scenario when only a small dataset is available, which is the most common case for railway data. The reason behind this stems from the low number of publicly available datasets with photos of the tracks. As a result, it is necessary to rely on a small number of public images or to gather new photos. The process of preparing new training datasets with railway images is costly and time-consuming since the images have to be labeled by experts with domain knowledge. This increases the need for precise upfront estimation of how many images are needed to obtain an accurate machine learning model. In this article, we show how much data are enough to train a neural network that detects track components and which architectures are best for this task. The key contributions of this paper are as follows:

- We conducted a benchmark to determine the sufficient data volume for railway component detection. We have shown how the YOLO and MobileNet neural network architectures perform for different sizes of datasets. We have used a completely new dataset with track images we collected and labeled. The results of the analysis will be valuable to anyone designing their own railway dataset, as we provide an estimate of the sufficient size of the data.
- We introduced a novel method of extracting background images (BIE) that can be used to enrich the datasets for the railway object detection task. We have shown that this method allows us to obtain better neural networks for really small datasets. BIE is useful to improve the performance of any models for railway track object detection.

This paper is organized as follows. Section 2.1 describes railway track and its components, Section 2.2 gives an overview of machine learning algorithms for OD, Section 2.3 introduces our novel Background Image Extraction method, and Section 2.4 exhibits the details of the OD benchmark. Section 3 outlines the main results of the benchmark. Section 4 summarizes the findings presented in the paper.

## 2. Materials and Methods

### 2.1. Railway Track

In this Section, we describe the railway track components that are detected in experiments in Section 2.4—rails, sleepers, and fasteners. The image areas without the mentioned components mostly contain track ballast. For the object-detection task, we consider ballast part of image background. The added examples are the images used in experiments, so they illustrate the data used in model training.

**Figure 1:**
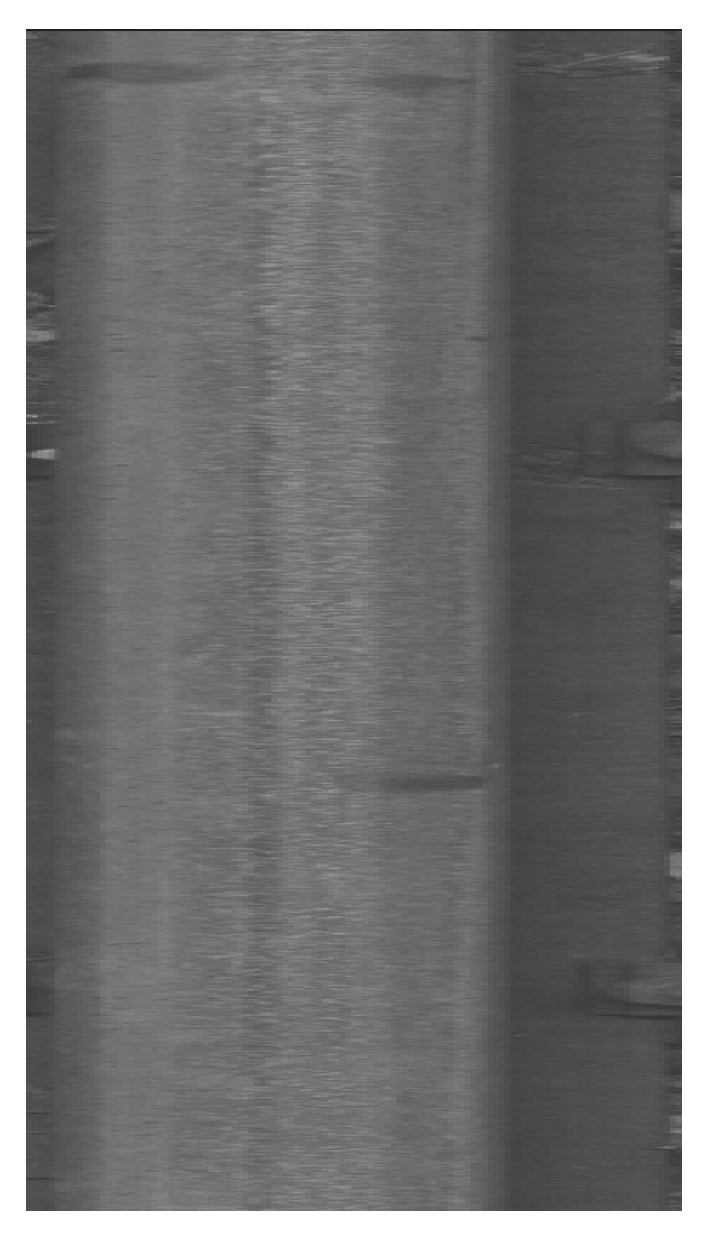
Example of rail.

**Figure 2:**
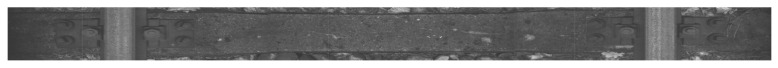
Example of concrete sleeper.

**Figure 3:**
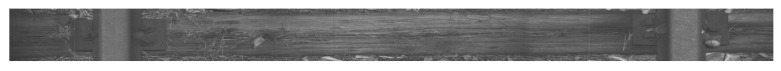
Example of wooden sleeper.

**Figure 4:**
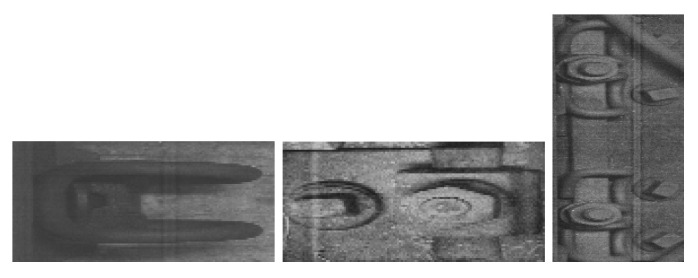
Examples of different types of fasteners.

**Figure 5:**
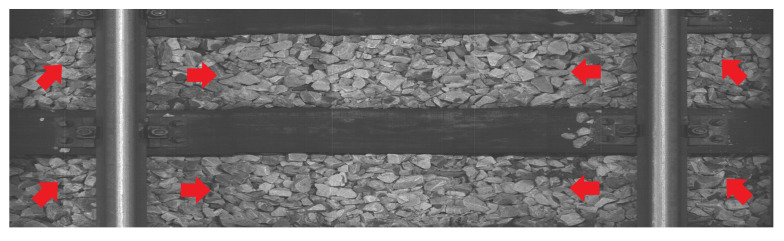
Example of railway track with marked track ballast.

Rails are steel bars that are the surface on which trains can move. Figure 1 shows an example of rail used on a railway track. Sleepers serve as support for rails, fixing them in position. Figure 2 and Figure 3 show examples of concrete and wooden sleepers. Fasteners are elements used to keep rails fastened to sleepers. Figure 4 shows examples of different types of fasteners on railway tracks. Track ballast is defined as a layer of crushed materials, usually rocks, placed around sleepers. Figure 5 shows a railway track with red arrows pointing to the ballast.

### 2.2. Machine Learning Models for Object Detection

Object detection is a computer vision technique for locating objects with bounding boxes (bboxes). Nowadays, convolutional neural networks (CNN) perform very well in this task. As a result, there is plenty of research on various architectures, for example, region-based convolutional neural networks (R-CNN) [29]. The idea of R-CNN is to start with a selective search [30]—a region-proposal procedure to pick out regions in the image that may contain objects of interest. In the next step, CNN extracts a feature vector from each region proposal, then a classification model assigns classes and scores to the extracted vectors. In the last step, a non-maximum suppression algorithm rejects image proposals with large intersection over union (IoU) overlap with higher-scored image proposals.

While R-CNN achieves satisfactory results, the drawback of this approach is the speed of training and prediction. To overcome these issues, Faster R-CNN [31] replaces the use of selective search with CNN. As a result, Faster R-CNN takes an entire image and processes it through a region-proposal network and then through a neural network that predicts classes of objects. This increases the detection speed, yet the prediction time is still not fast enough for real-time applications.

The Single-Shot Detector (SSD) is one of the fastest ways to achieve accurate object detection [32]. SSD consists of a feature-extractor backbone and SSD head. A backbone is a pre-trained classification neural network with a removed fully connected classification layer. The SSD head consists of convolutional layers added to the backbone to find the most appropriate bounding boxes. There is also a mobile variant of SSD, SSDLite [33], where regular convolutions in the SSD head are replaced with separable convolutions, which reduces both the parameter count and the computational cost compared to regular SSD. In Section 2.4, we used MobileNetV3-small as a backbone feature extractor in SSDLite, which is the same combination that the authors of MobileNetV3 used in their benchmarks Section 2.4. The MobileNet architecture is based on depth-wise separable convolutions that reduce the number of parameters [34]. In MobileNetV2, the authors introduced new inverted residual blocks [33] and in MobileNetV3 they added squeeze and excitation layers [35]. The MobileNetV3-small architecture is a variant targeted to low-resource use cases and we have chosen it for experiments because of its low number of parameters, which assures their ability to catch relationships in the data based on a small number of samples.

Another object-detection architecture is You Only look Once (YOLO) [36,37], which has become very popular in recent years. YOLOv5 is composed of three parts: backbone, neck, and detection networks. The backbone CNN aggregates image features that are processed in the neck network, creating Feature Pyramid Networks [38]. Finally, the detection network predicts each object’s class, probability, and bbox position. In experiments in Section 2.4, we have used two small YOLOv5 variants, the smallest variant nano (YOLOv5n) and variant small with ghost bottleneck (YOLOv5s-ghost). The small number of parameters means that the model has a chance to perform well on the low-volume datasets that are typical for railway OD. Moreover, YOLO’s good performance in a wide variety of applications implies that this architecture has great potential for railway applications as well.

We measured models’ performance with mean average precision (mAP) and mean average recall (mAR) [39]. Precision measures how well a model finds true positives and recall measures the proportion of true positive predictions,

(1) $$Precision_{t}=\frac{TP_{t}}{TP_{t}+FP_{t}},$$

(2) $$Recall_{t}=\frac{TP_{t}}{TP_{t}+FN_{t}},$$

where $TP_{t}$ denotes the number of true positives, $FP_{t}$ denotes number of false positives, and $FN_{t}$ denotes the number of false negatives. The value of *t* determines the IoU overlap above which bboxes are considered to be the same; thus, if the IoU value for predicted and true bboxes is greater than *t*, the predicted bbox is considered to be correct.

The average precision is the area under the precision–recall curve obtained by plotting the precision and recall values as a function of model’s confidence. The mAP@t is the mean of the average precision values over all classes with a given IoU overlap threshold *t*. For example, mAP@0.5 is the mean average precision for an IoU overlap threshold *t* equal to 0.5. In the experiments, we use also mAP@[0.5,0.95], which is an average of the mAP values for different IoU thresholds, starting from 0.5 and finishing at 0.95 with a step of 0.05.

The average recall is the area over a recall–IoU threshold for IoU ∈ [0.5, 1] and mAR is the mean of the average recall across all classes. mAR n denotes that mAR is calculated based on the top *n* bboxes detected in the image. In the experiments we have used an mAR of 100.

### 2.3. Background Image Extraction

**Figure 6:**
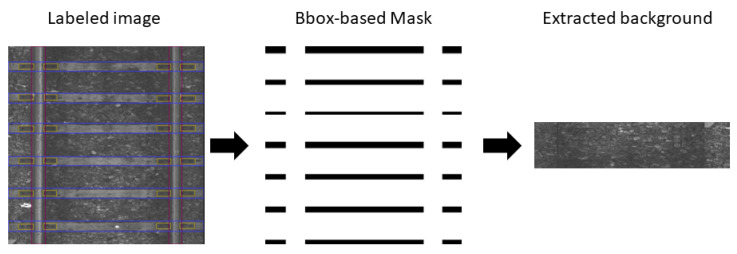
A diagram of the BIE method.

In this Section, we describe our novel method, named Background Image Extraction (BIE), which cuts out areas without bboxes from the railway photo and then joins them into a new image. Adding background images to the training set is a common procedure to improve model performance—the same stands for detecting railway components where the background consists mostly of ballast. Adding background images with no objects to detect to the training phase allows neural networks to learn what they should avoid detecting, which improves the performance of their predictions. Due to the distinctive composition of the railway track, we came up with a railway-targeted method of extracting background images. Figure 6 shows the general idea behind BIE. A bbox-based mask is extracted based on a labeled image based on the position of the track component. Then, a mask is used to cut out areas in the image that are merged into a new background image.

**Figure 7:**
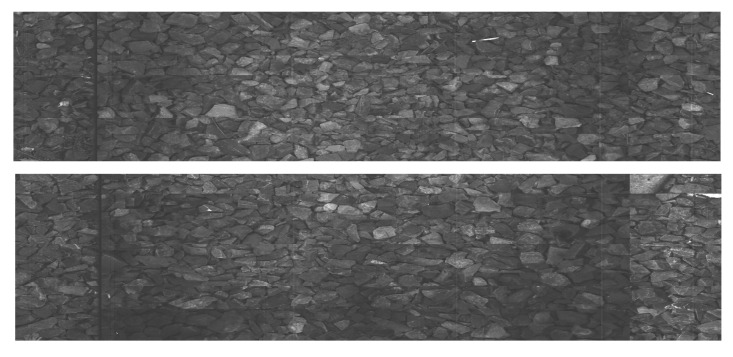
Example backgrounds extracted with BIE.

Algorithm 1 shows the procedure of mask extraction; its result is an array of the same size as an input image. In the mask, values of 0 represent the background and 225 non-background areas. Initially, each pixel within the mask has a value of 0. To identify the background, i.e., the area with ballast only, bboxes of rails are pulled to the top and bottom edges of the image, while bboxes of fasteners and sleepers are pulled to the side edges of the image. Then, the area of the pulled bboxes is treated as non-background and cropped out by setting the values of the corresponding pixels to 255. Such an approach ensures that after cropping, the union of the remaining parts will form a rectangle that contains only ballast. The newly created background image can then be used to enhance the training dataset. Figure 7 shows example backgrounds extracted with BIE.
**Algorithm 1** Mask extraction from railway image labeled with bboxes. The dot denotes a reference to the bbox property; thus, bbox.label means a class of bbox, bbox.width and bbox.height are its width and height, Additionally bbox.x_left and bbox.y_top denote the x coordinate of the left edge and y coordinate of the top edge of the bbox, respectively.**Require:** n: image width, m: image height, x_margin: x coordinate bbox margin used for mask extraction, y_margin: y coordinate bbox margin used for mask extraction
image_mask ← zeros(n, m)                           ▹ An array of size n × m filled with zeros.
**for** bbox in bboxes **do**
      **if** bbox.label is “rail” **then**
          x_left = bbox.x_left − x_margin
            **if** x_left < 0 **then**              x_left ← 0
          **end if**
          box_width ← bbox.width + x_margin ∗ 2
          y_top ← 0                                  ▹ Extend rail to whole image height.          box_height ← bbox.height
      **else**
          x_left ← 0                            ▹ Extend non-rail elements to whole image width.          box_width ← n
          y_top ← bbox.y_top − y_margin
          **if** y_top < 0 **then**                y_top ← 0
          **end if**
          box_height ← bbox.height ∗ n + y_margin ∗ 2
      **end if**
                                    ▹ Set area in mask related to the adjusted bbox to 255.
      image_mask[top_y: top_y + box_height, top_x: top_x + box_width] = 255
**end for**

All background images used in the experiments in Section 2.4 were created with BIE with x_margin 30 px and y_margin 90 px. Backgrounds with a width or height smaller than 100 px were filtered out.

### 2.4. Experiment

The aim of the experiment is to establish a sufficient data volume to train an efficient railway object detection model. Railway datasets usually are small and consist of images that are similar to each other. Moreover, infrastructure objects are also similar and are of a regular, rectangular shape. To find the number of images sufficient to obtain an accurate detector, we prepared training subsets of different sizes and trained the most common object-detection models. The training was carried out with and without the background-extraction method.

#### 2.4.1. Data Acquisition and Dataset

**Figure 8:**
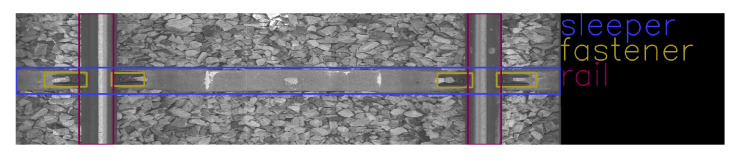
Example of a labeled short image of size 2083 px × 500 px with concrete sleepers.

**Figure 9:**
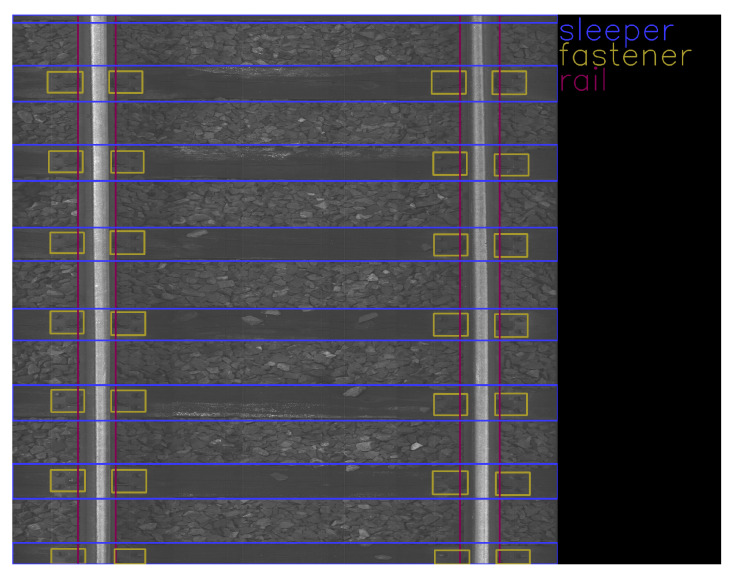
Example of a labeled long image of size 2083 px × 2100 px with wooden sleepers.

The data were collected on 2 March 2022 and 13 April 2022 at Warszawa Grochów motive power depot in Poland. All images are grayscale and come from line-scan cameras (raL4096-24gm - Basler racer, Basler AG, Ahrensburg, Germany). placed on the draisine running on the railway tracks. The photos contain track sections with wooden and concrete sleepers and do not contain switches. The dataset consists of 348 labeled images, including 299 short images of size 2083 px × 500 px and 49 long images of size 2083 px × 2100 px. Rails, sleepers, and fasteners are labeled on the images with bboxes. Figure 8 and Figure 9 show the annotations of short and long photos, respectively.

#### 2.4.2. Experiment Settings

The experiments were conducted on an AMD Ryzen 5 4600H CPU (Advanced Micro Devices, Inc., Santa Clara, California, United States) with Radeon Graphics (3.00 GHz, 32 GB RAM) and an NVIDIA GeForce RTX 2060 (CUDA version 12.0) (Nvidia Corporation, Santa Clara, California, United States) with Python 3.8.10 in the 64-bit Windows 10 business operating system.

We split the dataset into training, validation, and testing subsets of sizes 300, 24, and 24. We then took subsets of the training set of sizes 240, 180, 120, 90, 60, and 30, where each successive subset is contained in the preceding subset. The sizes of all subsets, along with the number of extracted backgrounds, are in Table 1.

We compared three neural networks: YOLOv5n, YOLOv5s-ghost, and MobileNetV3-small. Since the characteristic of the railway OD task is the small data volume, we have chosen architectures that have a small number of parameters and therefore, do not require much training data and have a fair chance to fit well. The models were trained for 100 epochs with default hyperparameters on all training subsets, both with and without extracted backgrounds, a total of 14 datasets. Detailed information about the hyperparameter values is in Table 2. For each training subset, the best model across all epochs was chosen based on its performance on the validation subset, then the final model performances were computed on the testing subset with mAP@0.5, mAP@[0.5, 0.95], and mAR 100.

Changes in the hyperparameter values listed in Table 2 influence the model predictions and performance. For a smaller number of epochs, the models might not be able to learn relationships in the data and therefore, might achieve a poor quality on both the training and testing subsets. For a larger number of epochs, the models will learn the data better, but there is a risk of overfitting to the training data and poor generalization. Setting a higher value of learning rate causes a faster loss decrease but increases the risk of missing the optimal minimum. For the lower value of learning rate, the decrease in the loss is lower; therefore, training will take longer and there is a risk of falling into a local minimum. Resizing images to a smaller size will lead to their poor quality; some details can be missed and therefore, the model will not be able to detect objects properly. Too-large image sizes will make it harder for models to properly fit the data when there is a small number of images in the training subset.

All background images used in the experiments in Section 2.4 were created with BIE with the hyperparameter values described in Table 3. Backgrounds with a width or height smaller than 100 px were filtered out.

## 3. Results

Table 4, Table 5 and Table 6 show the results of YOLOv5n, YOLOv5s-ghost, and MobileNetV3-small on the testing subset. The YOLOv5n architecture achieved the best performance in terms of all performance measures; its variant trained on datasets with background extraction performed the best or not far below the best result. YOLOv5s-ghost and MobileNetV3-small performed significantly worse in terms of mAP@0.5, mAP@[0.5, 0.95], and mAR. This illustrates an advantage of the YOLOv5n model as a railway object detector, which is the smallest of the neural networks taken into consideration. The task is relatively simple and the dataset so small that the large number of features in more capacious architectures caused overfitting.

**Figure 10:**
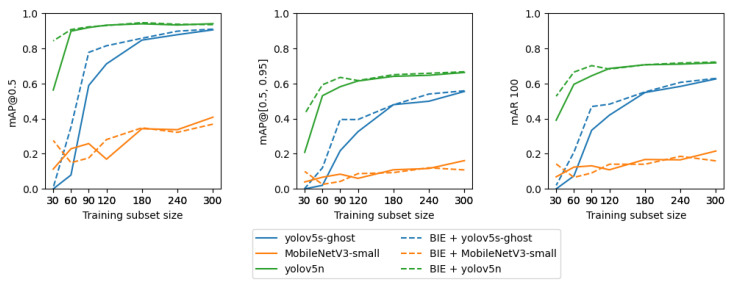
The relationship between the size of the training subset (x-axis) and model performance on the corresponding testing subset (y-axis). Each plot corresponds to a different performance measure. Colors mark neural network architectures and line types mark the presence of background images extracted with BIE in the training subset. This plot is the visualization of Table 4, Table 5 and Table 6.

Figure 10 shows the relationship between the training subset size and model performance. The plot shows that for training subsets that consist of 120 or fewer observations, YOLO models trained with BIE performed better than their variants trained without BIE. For training subsets containing more than 120 observations, there was no noticeable improvement of the YOLOv5n model trained with BIE when compared to its variant trained without BIE. Nevertheless, including background images in training subsets larger than 120 observations improved YOLOv5s-ghost. MobileNetv3-small did not show a noticeable quality increase after adding background images. The plots show that for training subsets consisting of more than 120 observations, the relative improvement of YOLOv5n’s performance is slight. Therefore, 120 is a sufficient number of observations to train an accurate model. In addition, when BIE is applied, even 90 observations are enough to achieve the same performance as 120 observations without BIE. YOLOv5n, compared to YOLOv5s-ghost and MobileNetv3-small, consists of a smaller number of parameters; therefore, it is expected to require fewer observations to achieve a good performance, which is consistent with the experimental results. BIE augments the training set with additional background images to teach algorithms which objects should not be detected and it can be seen that for small datasets it does indeed improve the quality of YOLO models. In contrast, it does not improve the quality of MobileNet, presumably because the model consists of the largest number of parameters and needs a larger volume of data.

**Figure 11:**
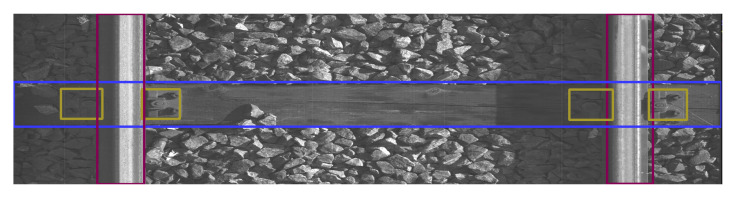
Example BIE + YOLOv5n model prediction for short image.

**Figure 12:**
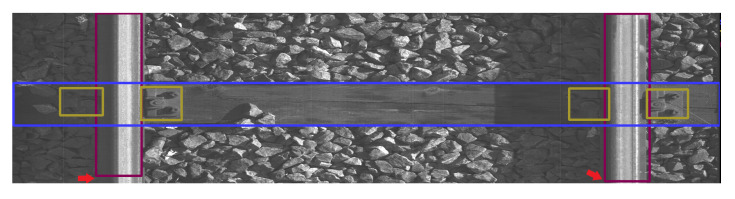
Example BIE + YOLOv5s-ghost model prediction for short image with marked incorrectly detected bboxes.

**Figure 13:**
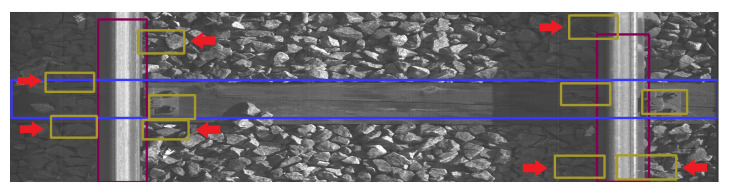
Example BIE + MobileNetV3-small model prediction for short image with marked incorrectly detected bboxes.

**Figure 14:**
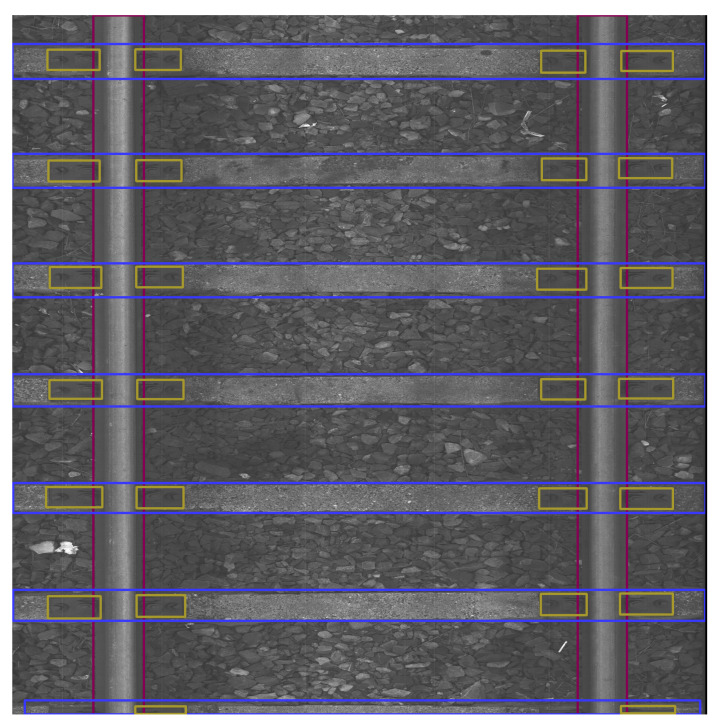
Example BIE + YOLOv5n model prediction for long image.

**Figure 15:**
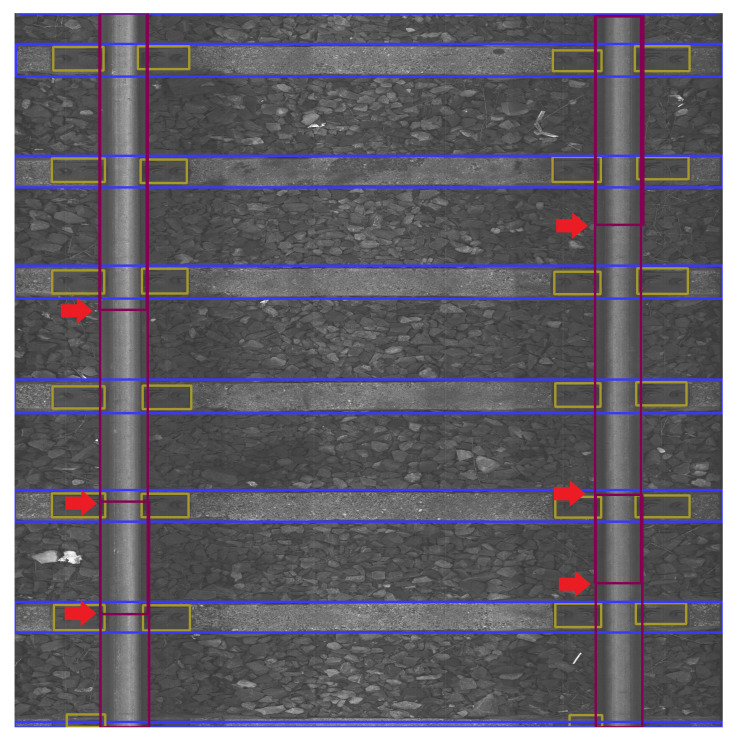
Example BIE + YOLOv5s-ghost model prediction for long image with marked incorrectly detected bboxes.

**Figure 16:**
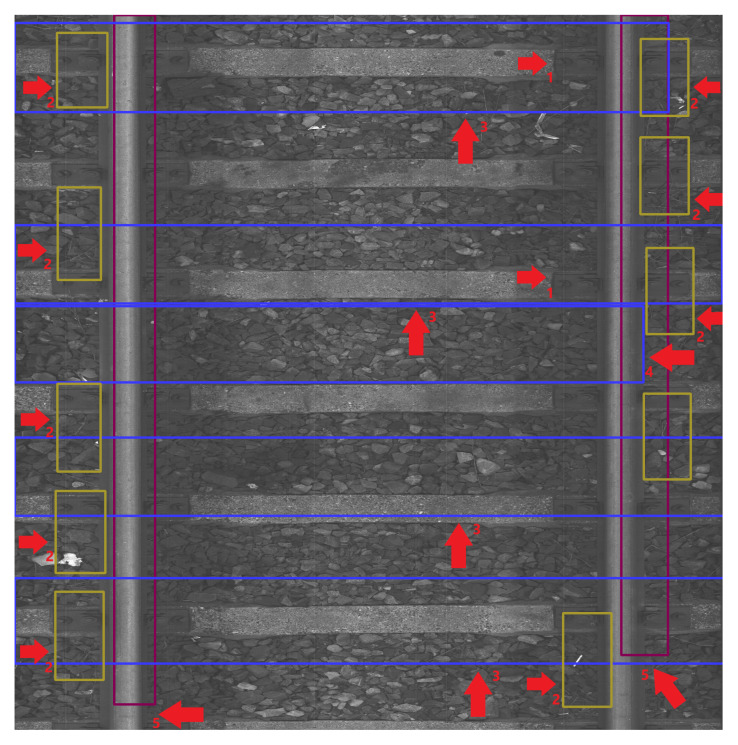
Example BIE + MobileNetV3-small model prediction for long image with marked incorrectly detected bboxes.

To showcase the phenomena observed throughout the entire test set, we present a sample of images. Figure 11, Figure 12, Figure 13, Figure 14, Figure 15 and Figure 16 show the representative visualizations of example railway component detections from the testing subset. All figures contain the results of models trained on datasets with Background Image Extraction. The red arrows and numbers on figures point to incorrectly detected bboxes. YOLOv5n returns more accurate detections when compared to YOLOv5s-ghost and MobileNetv3-small. A comparison between Figure 11 and Figure 12 and Figure 14 and Figure 15 shows that YOLOv5s-ghost detects sleepers and fasteners as well as YOLOv5n, but incorrectly detects rail bboxes, which are too short (Figure 12) and overlapping (Figure 15). In turn, Figure 13 shows that on short images MobileNetV3-small detects additional fasteners in inaccurate places, which may be caused by the fact that MobileNetV3 is a backbone for SSDLite, which is a variant of Single-Shot Detector (SSD) and SSD is known for worse detections on small objects [40]. Figure 16 shows that on long images MobileNetV3-small detects too-large bboxes for fasteners and sleepers, which might be caused by the small number of long images in the dataset and therefore, the model has overfitted the short images.

The red arrows in Figure 12 point to incorrectly detected rail bboxes—they are too short.

The red arrows in Figure 13 point to fastener bboxes that are detected in incorrect places.

The red arrows in Figure 15 point to places where the detected rail bboxes are overlapping.

The red arrows in Figure 16 with the number 1 point to examples of undetected fastener bboxes, with number 2 to detected fastener bboxes that are too large, with number 3 to detected sleeper bboxes that are too large, with number 4 to a detected sleeper bbox that is in the incorrect place, and with number 5 to detected rail bboxes that are too short.

## 4. Discussion

In summary, the experiments show that the task of railway component detection is relatively simple, and a training set consisting of 120 labeled observations is sufficient to train an efficient model. In addition, the results show that BIE may enrich a small dataset and reduce the number of observations needed to train an accurate model from 120 to 90. The model that performed best is YOLOv5n, which is the smallest of the considered architectures, supporting the hypothesis that the task is simple and does not require much labeled data.

## 5. Conclusions

In this paper, we searched for a sufficient data volume for the detection of railway infrastructure components. As a result of this study, the following findings have been made:

- In total, 120 training observations are enough to train an efficient YOLO model. At the same time, the authors of YOLO recommend using over 1500 images per class and over 10000 labeled objects for best training results (https://github.com/ultralytics/yolov5/wiki/Tips-for-Best-Training-Results), accessed on 3 July 2023, which is approximately 100 times more than was needed in our experiment. Taking this into account, a sufficient detector of the railway objects requires a relatively small amount of data, which is desirable since labeled railway images are not easily available;
- The number of observations required to train an efficient railway OD model can be reduced to 90 observations after applying our method BIE, which allows for background extraction from the training subset. The use of background images is common in OD tasks since backgrounds are usually simple to acquire, which is different for railway backgrounds, which cannot have any images that do not contain railway components. These should be photos composed of the ballast alone, which requires additional effort to obtain them. Thus, this paper’s result that BIE can be used to extract backgrounds from training images is an important finding;
- The best model for the railway object detection task is YOLOv5n, which is the smallest of the YOLO models, and therefore, is more robust for overfitting to small datasets.

In summary, this paper’s results demonstrate the great potential of neural networks for detecting railway infrastructure objects. With a limited amount of data labeling, it is possible to obtain adequate models that can support people in railway track condition analysis.

## Figures and Tables

**Table 1:** Dataset splits for experiment with numbers of observations.

Split	Number of Full Railway Images	Number of Background Images Extracted with BIE	Total Number of Images
training subset 30	30	12	42
training subset 60	60	25	85
training subset 90	90	34	124
training subset 120	120	49	169
training subset 180	180	69	249
training subset 240	240	86	326
training subset 300	300	106	406
validation subset	24	7	31
testing subset	24	-	24

**Table 2:** Values of models’ hyperparameters.

Hyperparameter	YOLOv5n	YOLOv5s-Ghost	MobileNetV3-Small
Number of epochs	100	100	100
Batch size	16	16	32
Image size (in pixels)	640 × 640	640 × 640	320 × 320
Learning rate	0.001	0.001	0.01

**Table 3:** Values of BIE hyperparameters.

Hyperparameter	Value
x_margin	30 px
y_margin	30 px
Background width or height minimal size	100 px

**Table 4:** Values of mAP@0.5 on testing subset. Bold values are the highest ones for each size of the training subset. mAP@0.5 measures how well a model finds true objects on the image, allowing a 50% margin of error for the bbox area.

Method	30 obs.	60 obs.	90 obs.	120 obs.	180 obs.	240 obs.	300 obs.
YOLOv5n	0.563	0.899	0.919	**0.933**	0.941	0.934	**0.942**
BIE + YOLOv5n	**0.843**	**0.908**	**0.923**	0.931	**0.947**	**0.938**	0.936
YOLOv5s-ghost	0.00	0.079	0.589	0.712	0.848	0.879	0.906
BIE + YOLOv5s-ghost	0.008	0.352	0.778	0.816	0.858	0.899	0.910
MobileNetV3-small	0.113	0.229	0.258	0.169	0.343	0.337	0.408
BIE + MobileNetV3-small	0.276	0.149	0.176	0.281	0.348	0.322	0.369

**Table 5:** Values of mAP@[0.5, 0.95] on testing subset. Bold values are the highest ones for each size of the training subset. mAP@[0.5, 0.95] measures how well a model finds true objects in the image, averaging over different margins of error for the bbox area.

Method	30 obs.	60 obs.	90 obs.	120 obs.	180 obs.	240 obs.	300 obs.
YOLOv5n	0.208	0.531	0.582	0.615	0.641	0.647	0.663
BIE + YOLOv5n	**0.427**	**0.593**	**0.636**	**0.617**	**0.651**	**0.659**	**0.667**
YOLOv5s-ghost	0.00	0.020	0.218	0.326	0.480	0.499	0.555
BIE + YOLOv5s-ghost	0.002	0.120	0.395	0.395	0.479	0.541	0.559
MobileNetV3-small	0.039	0.067	0.084	0.060	0.109	0.116	0.161
BIE + MobileNetV3-small	0.099	0.026	0.042	0.087	0.093	0.120	0.108

**Table 6:** Values of mAR 100 on testing subset. Bold values are the highest ones for each size of the training subset. mAR 100 measures the proportion of the top 100 correctly detected objects to all objects in the image.

Method	30 obs.	60 obs.	90 obs.	120 obs.	180 obs.	240 obs.	300 obs.
YOLOv5n	0.391	0.596	0.645	**0.686**	**0.707**	0.711	0.718
BIE + YOLOv5n	**0.528**	**0.666**	**0.702**	0.683	**0.707**	**0.717**	**0.723**
YOLOv5s-ghost	0.00	0.074	0.334	0.420	0.549	0.584	0.626
BIE + YOLOv5s-ghost	0.020	0.208	0.469	0.483	0.552	0.607	0.630
MobileNetV3-small	0.069	0.124	0.132	0.109	0.167	0.165	0.216
BIE + MobileNetV3-small	0.142	0.065	0.091	0.140	0.140	0.185	0.160

## Data Availability

Data unavailable for public sharing due to privacy reasons.

## Abbreviations

The following abbreviations are used in this manuscript:

bbox	Bounding Box
BIE	Background Image Extraction
CNN	Convolutional Neural Network
IoU	Intersection Over Union
KNN	K-Nearest Neighbors
ML	Machine Learning
mAP	Mean Average Precision
mAR	Mean Average Recall
OD	Object Detection
R-CNN	Region-based Convolutional Neural Networks
SSD	Single-Shot Detector
SVM	Support Vector Machines
YOLO	You Only Look Once
YOLOv5n	YOLO version 5 nano
YOLOv5s-ghost	YOLO version 5 small with ghost bottleneck
